# Development of an Advanced Multicellular Intestinal Model for Assessing Immunomodulatory Properties of Anti-Inflammatory Compounds

**DOI:** 10.3389/fphar.2021.639716

**Published:** 2021-04-16

**Authors:** Diego Marescotti, Giuseppe Lo Sasso, Diego Guerrera, Kasper Renggli, Pedro A. Ruiz Castro, Romain Piault, Vincent Jaquet, Fabian Moine, Karsta Luettich, Stefan Frentzel, Manuel C. Peitsch, Julia Hoeng

**Affiliations:** PMI R&D, Philip Morris Products S.A., Neuchâtel, Switzerland

**Keywords:** immune competent cells, inflamed intestine, nicotine, inflammatory bowel disease, intestinal inflammation, *in vitro* co-culture, tobacco alkaloids, anatabine

## Abstract

Intestinal inflammation is the collective term for immune system-mediated diseases of unknown, multifactorial etiology, with often complex interactions between genetic and environmental factors. To mechanistically investigate the effect of treatment with compounds possessing immunomodulating properties in the context of intestinal inflammation, we developed an immunocompetent *in vitro* triculture intestinal model consisting of a differentiated intestinal epithelial layer (Caco-2/HT29-MTX) and immunocompetent cells (differentiated THP-1). The triculture mimicked a healthy intestine with stable barrier integrity. Lipopolysaccharide treatment triggered a controlled and reversible inflammatory state, resulting in significant impairment of barrier integrity and release of pro-inflammatory cytokines and chemokines, which are known hallmarks of intestinal inflammation. Treatment with known anti-inflammatory reference compounds (TPCA-1 and budenoside) prevented the induction of an inflammatory state; the decreasing triculture responses to this treatment measured by cytokine release, transepithelial electric resistance (TEER), and epithelial layer permeability proved the suitability of the intestinal model for anti-inflammatory drug screening. Finally, selected tobacco alkaloids (nicotine and anatabine (*R*/*S* and *S* forms)) were tested in the *in vitro* triculture for their potential anti-inflammatory properties. Indeed, naturally occurring alkaloids, such as tobacco-derived alkaloids, have shown substantial anti-inflammatory effects in several *in vitro* and *in vivo* models of inflammation, gaining increasing interest. Similar to the anti-inflammatory reference compounds, one of the tobacco alkaloids under investigation partially prevented the decrease in the TEER and increase in permeability and reduced the release of pro-inflammatory cytokines and chemokines. Taken together, these data confirm that our *in vitro* model is suitable for screening potential anti-inflammatory compounds in the context of intestinal inflammation.

## Introduction

The gastrointestinal tract is composed of an epithelial layer, which is exposed to an overwhelming load of foreign antigens arising from commensal microorganisms, dietary products, and occasional pathogens (Tilg and Moschen, 2015). In the physiological state, the gastrointestinal mucosa forms a unique immunological environment in which tolerance to commensal microbes and/or food antigens and specific immunity to pathogens coexist in a fine-tuned immune equilibrium (Schenk and Mueller, 2008; Honda and Littman, 2012; Tilg and Moschen, 2015). When this tightly regulated immune homeostasis becomes altered, pathological inflammation and disruption of the epithelial barrier might occur (Peterson et al., 2015). The resulting intestinal inflammation is becoming increasingly relevant as one of the more commonly occurring diseases in developed countries (Ng et al., 2017). The indications range from irritable bowel syndrome to chronic inflammatory conditions of the gut related to a combination of genetic, environmental, and immunological factors that impact on normal host–microbe interactions. (Abraham and Medzhitov, 2011; Kaistha and Levine, 2014; Atreya and Neurath, 2015; Ramos and Papadakis, 2019). The etiology of intestinal inflammation remains poorly understood; thus, development of new approaches for predicting disease evolution and personalized response to treatment are of primary clinical relevance (Molodecky et al., 2012; Ponder and Long, 2013). Although no unique cause has been determined for inflamed intestine, environmental factors, including medication, stress, diet, and smoking, are known to impact disease onset and progression (Abraham et al., 2017).

Naturally occurring alkaloids derived from plants or medicinal herbs gained much interest as a potential treatment against inflamed intestine due to their significant antioxidant and anti-inflammatory effect (Niu et al., 2015; Wu et al., 2018; Peng et al., 2019). These natural derived bioactive compounds can be sourced from living organisms, such as bacteria, fungi, plants, and animals (Moreira et al., 2018). Plant-derived alkaloids have been shown to possess anti-inflammatory properties as demonstrated in animal models of disease, including inflammatory bowel disease (IBD), a group of disorders characterized by chronic inflammation of the gastrointestinal tract (Niu et al., 2013; Chen et al., 2017; Ruiz Castro et al., 2020). The positive effects of alkaloid have been linked to a modulation of intestinal oxidative stress (Karatay et al., 2017; Rodrigues de Carvalho et al., 2018), a strengthening of the epithelial barrier function (Zhang et al., 2017), and a boost of gut microbiota (Zhang et al., 2018).

Among the most studied plant-derived alkaloids are pyridine alkaloids derived from the tobacco plant (*Nicotiana tabacum*) (Saitoh et al., 1985). Nicotine—the major alkaloid in tobacco—as well as all minor tobacco alkaloids have been shown to be pharmacologically active (Clark et al., 1965). Nicotine is addictive and not risk free. Minors, pregnant or breast-feeding women, and people with heart disease, severe high blood pressure or diabetes should not use tobacco or nicotine containing products (McNeill et al., 2018). However, several *in vitro* and *in vivo* studies that mimicked intestinal inflammation have supported the notion of nicotine-dependent anti-inflammatory effects (Cloëz‐Tayarani and Changeux, 2007; McGilligan et al., 2007; Verschuere et al., 2012; Hayashi et al., 2014), which might be mediated by the activation of nicotinic acetylcholine receptors (nAChR) and the consequent activation of cholinergic anti-inflammatory pathways (Wang et al., 2003; Ulloa, 2005). However, the contrasting results obtained by both *in vivo* and clinical studies on the role of nicotine in the context of intestinal inflammation (AlSharari et al., 2013; Gomes et al., 2018), as well as the high frequency of nicotine-related adverse events observed in those clinical studies (Lunney and Leong, 2012), triggered the investigation of other minor tobacco-alkaloids (Olsson et al., 1993; Bai et al., 2007; Paris et al., 2013a; Paris et al., 2013b).

To support the investigation of compounds with potential anti-inflammatory properties in the gastrointestinal system, we developed an immunocompetent *in vitro* intestinal model that recapitulates the intestinal barrier and contains a functional mucus layer and immunocompetent cells. Thus, we developed a tri-culture model using the classical Transwell® configuration, in which epithelial cells, such as Caco-2 and HT29-MTX, are seeded in the apical compartment, while the immune cells, THP-1, are added to the basolateral side.

The epithelial cell line Caco-2 has been the most widely used intestinal cell line model for interrogating various endpoints, including intestinal absorption, cell membrane permeability, passive and active diffusion of drug molecules, and inflammatory response (Hidalgo et al., 1989; Hillgren et al., 1995; Cheng et al., 2008). Upon reaching full confluence, Caco-2 cells spontaneously differentiate over the following 3 to 4 weeks of culture and form a polarized cell monolayer, which is characterized by apical and basolateral membranes, tight junctions, and a brush border with microvilli on the apical side typical of human enterocytes (Chantret et al., 1988; Hidalgo et al., 1989). To better replicate the *in vivo* physiology of the intestine, we decided to coculture Caco-2 cells with a mucus-producing cell line, the stable clone HT29-MTX (Lesuffleur et al., 1990; Chen et al., 2010). Finally, to further complete the model and increase its relevance for anti-inflammatory compound assessment, we included the monocytic cell line THP-1 because of its potential for differentiation into macrophage- or dendritic cell-like cells. Finally, we established the triculture by using the classical Transwell® configuration, in which epithelial cells are seeded in the apical compartment, while immune cells are added to the basolateral side.

Although similar models have already been established (Leonard et al., 2010; Kaulmann et al., 2016; Susewind et al., 2016; Kampfer et al., 2017), we intended to design and obtain an *in vitro* model characterized by i) less laborious cell culture handling, ii) immune-to-epithelial cell pro-inflammatory crosstalk, and iii) functional readouts. As a proof of concept, after establishing and assessing the model with known anti-inflammatory drugs, we evaluated the putative anti-inflammatory effects of two well-known tobacco alkaloids, namely nicotine and anatabine. Therefore, using our model, we were able to investigate the crosstalk between intestinal epithelial cells and immune cells in the context of intestinal inflammation and its response to natural derived compounds.

## Materials and Methods

### Cell Lines

The human colon adenocarcinoma cell line Caco-2 (86010202, Sigma, St. Louis, MO, United States) was cultured in Eagle’s minimum essential medium (EMEM; 30-2003, ATCC, Manassas, VI, United States)) supplemented with 10% fetal bovine serum (FBS; 16140-071, Gibco, Gaithersburg, MD, United States), 1% non-essential amino acids (NEAA; 11140-050, Life Tech, Carlsbad, CA, United States), and 1% penicillin/streptomycin (P4333, Sigma). The human colon cell line HT29-MTX-E16 (86010202, Sigma) was cultured in Dulbecco’s modified Eagle’s medium (DMEM; D6546, Sigma) supplemented with 10% FBS (16140-071, Gibco), 1% NEAA (11140-050, Life Tech), 1% Glutamax (35050-061, Gibco), and 1% penicillin/streptomycin (P4333, Sigma). The human acute monocytic leukemia cell line THP-1 (Tib-202, ATCC) was cultured in Roswell Park Memorial Institute (RPMI) 1640 (R0883, Sigma) supplemented with 10% FBS (16140-071, Gibco), 1% Glutamax (35050-061, Gibco), and 1% penicillin/streptomycin (P4333, Sigma). The cells were tested for *mycoplasma* contamination and found negative.

### Triculture Assembly

Human Caco-2 and HT29-MTX cells (2 × 10^4^ in total) were seeded at a 9:1 ratio in 6.5-mm Transwell® with 0.4-µm pore polyester membrane inserts (Costar #3470) and allowed to grow and differentiate for 14 or 21 (depending on the aim of the experiment) days after full confluence in DMEM (Earle’s balanced salt solution) (M2279, Sigma) containing 2 mM glutamine (G7513, Sigma), 1% NEAA, 10% heat-inactivated FBS, and 1% penicillin/streptomycin. The culture medium (200 μL in the apical and 700 μl in the basolateral compartments) was replaced every 2 days. A day before the Transwell® containing epithelial cells was moved into the well containing THP-1 cells, the basolateral medium was replaced with 400 µL of RPMI. THP-1 cells (2.4 × 10^5^) were seeded in 600 µL of RPMI in 24-well plates and differentiated with 10, 20, or 40 ng/ml PMA (phorbol-12-myristate-13-acetate; P1585, Sigma) for ∼65 h. The PMA was removed after differentiation, and the cells were rested for 24 h. Then, the Caco-2/HT29-MTX-containing Transwell® insert was added into the well, with a basolateral medium volume of 400 µL. After assembly the triculture was rested for 24 h in preparation for pro-inflammatory induction and compound assessment.

### Histological Analysis

Histological sections were obtained from cultures harvested on day 12 of culture after the cells reached full confluence. The cultures were processed in accordance with previously published protocols (Iskandar et al., 2018; Zanetti et al., 2018). Briefly, the cultures were washed three times with PBS (phosphate-buffered saline; Merck, Darmstadt, Germany) and fixed for 2 h in 4% (w/v) paraformaldehyde solution in PBS (Thermo Fisher Scientific). After fixation, the cultures were washed three times in PBS and bisected; the resulting two pieces were placed in cassettes and processed with the Leica ASP300 S Tissue Processor (Leica Biosystems Nussloch GmbH, Nussloch, Germany). The processed cultures were embedded vertically and in parallel by using the MEDITE TES Valida Tissue Embedding System (MEDITE GmbH, Burgdorf, Germany), and 5-μm-thick sections were cut by using a Leica RM2255 microtome (Leica Biosystems Nussloch GmbH). The cut sections were mounted on Superfrost™ Plus slides (Thermo Fisher Scientific) and transferred to the Leica ST5020 automated stainer (Leica Biosystems Nussloch GmbH) for staining with hematoxylin, eosin, and Alcian blue. The stained slides were covered with coverslips automatically by using the Leica CV5030 automated coverslipper (Leica Biosystems Nussloch GmbH). Digital images of the stained slides were generated by using the NanoZoomer 2.0 slide scanner (Hamamatsu Photonics, Hamamatsu, Japan) at ×40 magnification.

### Immunofluorescence Staining

To detect goblet cells and tight junctions, we used antibodies targeting Muc5Ac and ZO-1, respectively. The cultures were first fixed with 4% (w/v) paraformaldehyde (Sigma, Saint Louis, United States) for 15 min and then blocked for 1 h in a blocking solution (0.5% Triton X-100, 5% normal goat serum, and 2% bovine serum albumin; all reagents from Thermo Fisher Scientific, Waltham, MA, United States) in 1× Dulbecco’s phosphate-buffered saline (D-PBS; without calcium, magnesium, or Phenol red; STEMCELL Technologies). The cultures were stained with a Muc5AC antibody conjugated to Alexa 550 (1:250, ab218714, Abcam) or a ZO-1 antibody (1:250, 339194, Thermofisher), diluted in D-PBS with 2% normal goat serum (Thermo Fisher Scientific, Waltham, MA, United States) and 1% bovine serum albumin (Thermo Fisher Scientific). Nuclei were counterstained by using ProLong™ Diamond Antifade Mountant with DAPI (4′,6-diamidino-2-phenylindole; Thermo Fisher Scientific). Images were acquired with the CellInsight™ CX7 HCS platform (Thermo Fisher).

### Chemicals

All compounds, including reference compounds, test compounds, and pro-inflammatory inducers, used in the study are reported in Supplementary Table S5.

### TEER

Cellular transepithelial electric resistance (TEER) was measured by using chopstick electrodes (STX-2) connected to an EVOM_Epithelial Voltohmmeter (World Precision Instruments, Berlin, Germany) after addition of 200 ml medium to the apical side of the tissues.

### WST-8 Cell Viability Assay

WST-8 bioreduction in single cell lines was measured by using Cell Counting Kit-8 (CCK-8, Sigma #96992). WST-8 is bioreduced by cellular dehydrogenases to an orange formazan product that is soluble in tissue culture medium; the amount of formazan produced is directly proportional to the number of living cells. WST-8 was added to the cells by diluting it 1:10 in the volume of medium present in the wells (i.e., 10–100 µL medium present in the 96-well plates for single cell lines, 20–200 µL medium present in the apical compartment of Transwell® inserts containing epithelial cocultures, or 40–400 µL medium present in the basolateral compartment containing the THP-1 cell part of the triculture). After 1 h of incubation at 37°C, 100 µL the medium was transferred to a 96-well plate and measured for absorbance at 450 nm by using a FluoStar Omega reader (BMG Labtech GmbH, Ortenberg, Germany). A blank consisting of WST-8 incubated with medium only was also added to the plate and subtracted from the measured absorbance values recorded from the samples.

### Permeability

Permeability was assessed by using FITC (fluorescein isothiocyanate)–dextran 4 kDa (FD4, Sigma-Aldrich) by adding 200 µL of growth medium (MEM) containing 1 mg/ml FD4 to the apical compartment. After a 1-h incubation at 37°C, 100 µl of basolateral medium was transferred to a 96-well plate (Sterilin black microtiter plates, 611F96BK, Thermo Fisher). Fluorescence intensity was measured at excitation and emission wavelengths of 400 and 535 nm, respectively, by using a FluoStar Omega reader. Fluorescence values from known serial dilutions of FD4 (0–5 μg/ml) were used to generate a standard curve. FD4 translocation from the apical to basolateral compartments was quantified by interpolation of fluorescence intensity values on the 4-parameter fit standard curve obtained. A blank consisting of cell culture medium was also added to the plate, and the corresponding values were subtracted from the measured concentrations of all samples. FD4 passage (in µg) was calculated by multiplying the values obtained with the volume present in the basolateral compartment (700 µl). Additionally, apparent permeability coefficient was calculated by using the following equation:

$$P_{app}=\left(\frac{dQ}{dt}\right)\times \left(\frac{1}{A\times C_{0}}\right)$$

defined as:

*P*_*app*_ = apparent permeability coefficient (cm/s)

*dQ*/*dt* [μg/s] = rate of appearance of FD4 on the basolateral side

*A* = surface area of the monolayers

*C*_0_ [μg/ml] = initial FD4 concentration in the apical compartment.

Fold change was calculated over the untreated control used as reference. Subsequently, data were normalized by setting the fold-change values of samples treated with only the inflammation inducer as 100% (reference). A *t*-test was performed for comparing cell cultures treated with the reference/test items as well as the inflammation inducer with those treated with only the inflammation inducer.

### Cytokine Measurement

Secreted cytokines were measured in medium collected from cell cultures treated with inflammation inducers or reference/test items along with inflammation inducers at various time points. Specific ELISA kits (interleukin (IL)-6, IL-8, tumor necrosis factor (TNF) α, and IL-1β) from R&D systems were used in accordance with the manufacturers’ instructions.

### Statistical Analysis

This study consisted of an exploratory phase and a confirmation phase, with the latter illustrated in Figures 4 and 5. Several independent experiments were performed only for the confirmation phase. For the exploratory phase, the technical replicates, generally different wells in a culture plate, were considered independently. Statistical differences between conditions were determined by two-tailed t-tests. Statistical equivalences between conditions were determined by two-one-sided *t*-tests (TOST) with a magnitude of region of similarity of ±20%. Statistical trends were determined by a fitting linear model. The *p* value for the linear model, *t*-test, and TOST were reported with asterisks: *p ≤ 0.05; **p ≤ 0.01; ***p ≤ 0.001; not significant (ns), *p* > 0.05. The *t*-test, linear model, and equivalence test were performed in R 3.5.1 with the t.test and lm functions from the stats package and the tost function from the equivalence package, respectively.

Datasets, detailed protocols, and additional data visualizations are available on the INTERVALS platform.

## Results

### Establishment of Caco-2/HT29-MTX Coculture and Evaluation of Pro-Inflammatory Response

Caco-2 and HT29-MTX, seeded at a 9:1 ratio (Hilgendorf et al., 2000; Chen et al., 2010), were cocultured over a period of 32 days and monitored for barrier integrity by evaluating the increase in TEER. TEER values reached a maximum of ∼900 Ω*cm^2^ 12 days after the cells reached full confluence (approximately 3 days after seeding), after which they remained stable in the range of 700–900 Ω*cm^2^ up to day 26 (Supplementary Figure S1A). Muc5AC and ZO-1 staining confirmed the presence of functional mucus-secreting HT29-MTX cells and establishment of tight junctions, respectively (Supplementary Figure S1B). Additionally, longitudinal slices were stained with hematoxylin, eosin, and Alcian blue for histological examination (Supplementary Figure S1C).

We first evaluated the effects of different pro-inflammatory inducers and the route of administration over 24 h after 21 days of maturation of the coculture. In particular, single concentrations of TNFα, IL-1β, and lipopolysaccharide (LPS) were added either apically or basolaterally on day 22, and TEER, membrane permeability, and basolateral IL-8 release were measured as pro-inflammatory readouts. Interestingly, none of the apical treatments affected membrane integrity, as no changes were observed in TEER (Supplementary Figure S2A) or membrane permeability (Supplementary Figure S2B) relative to the untreated control. IL-8 release, however, was increased when TNFα or IL-1β was applied apically (Supplementary Figure S2C). On the other hand, a marked decrease in TEER (Supplementary Figure S2D) together with an increase in membrane permeability (Supplementary Figure S2E) and IL-8 release (Supplementary Figure S2F) were observed when the coculture was treated basolaterally with TNFα. In contrast, basolateral IL-1β treatment showed only a weak effect on TEER and IL-8 release and no effect on membrane permeability (Supplementary Figures S2D,F,E, respectively). Of note, as previously documented, LPS treatment had no effect on any of the three readouts in the cells (Abreu et al., 2001).

**FIGURE 1:**
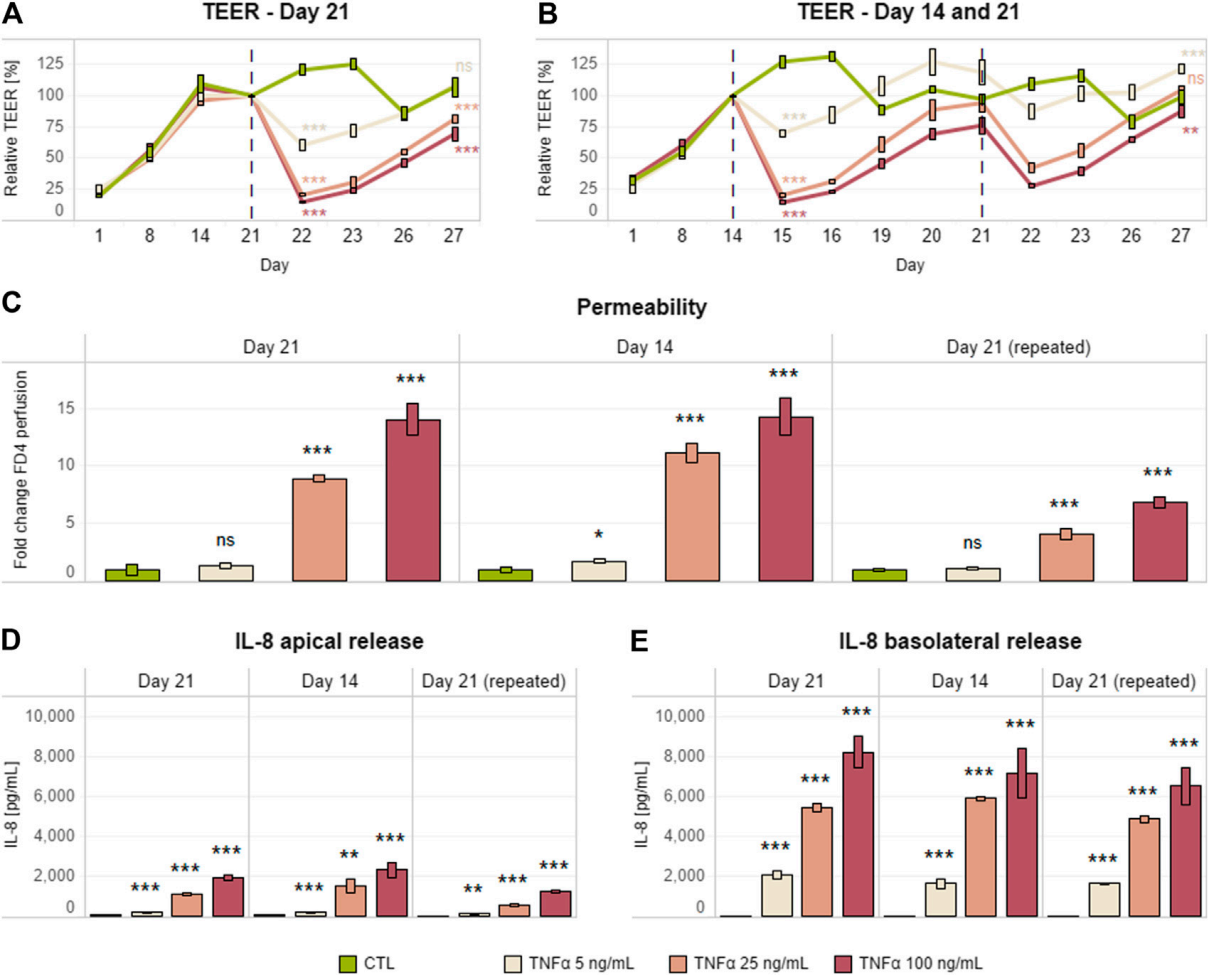
Single and repeated Caco-2/HT29-MTX pro-inflammatory induction after days 14 and 21. TEER **(A,B)**, permeability **(C)**, and IL-8 release **(D,F)** in a differentiated Caco-2/HT29-MTX coculture basolaterally treated with different concentrations of TNFα (0–100 ng/ml) on day 21 (single) or days 14 and 21 (repeated). Each inflammatory stimulus (red dashed line) was left for up to 24 h; this was followed by a recovery period of up to 120 h without an inflammatory inducer. TEER values are represented in percentage; zero-hour treatment was used as reference (100%). Permeability is expressed as fold change over the untreated control (CTL). Data are presented as the average of three technical replicates with the corresponding standard deviations (*n* = 1; m > 3). On the day at the end of the simulation and the last day recorded, statistical differences between the control without TNFα and each of the three concentrations of TNFα were determined by a two-tailed *t*-test, *p ≤ 0.05; **p ≤ 0.01; ***p ≤ 0.001; ns, *p* > 0.05. Equivalence comparisons and linear regression analysis are provided in Supplementary Tables S1 and S2. Abbreviations: TEER, transepithelial electrical resistance; TNF, tumor necrosis factor; CTL, control.

Although the generally accepted Caco-2 differentiation period is 21 days (Sambuy et al., 2005; Leonard et al., 2010; Pereira et al., 2015; Susewind et al., 2016), we evaluated the possibility of shortening the coculture incubation period from 21 to 14 days (Antunes et al., 2013) and still maintain an equivalent pro-inflammatory response. Differentiated Caco-2/HT29-MTX cocultures on day 14 or 21 were basolaterally treated with different concentrations of TNFα (5, 25, or 100 ng/ml) for 24 h. TNFα cause a dose-dependent decrease in TEER (Figures 1A,B) and increase in permeability (Figure 1C) and IL-8 release (Figures 1D,E), independent of the age of the coculture/duration of coculture maturation (Figures 1A,B). Upon removal of the pro-inflammatory stimulus, we also observed a time-dependent increase in TEER over the following 5 days.

We also tested a protocol involving two consecutive cycles of inflammatory induction and recovery on days 14 and 21. Thus, the Caco-2/HT29-MTX coculture treated on day 14 was further treated on day 21 with the same TNFα concentrations for an additional 24 h (Figure 1B). The dose–response outcomes of inflammatory induction on days 14 and 21 were similar in terms of TEER and permeability. However, the coculture appeared to be more resistant to the second stimulus, as the magnitude of effects on TEER and permeability was lower (Figures 1B–E).

Finally, a medium compatibility test was performed with a view toward assembly of the final triculture with THP-1 cells. The coculture medium (DMEM) in the basolateral compartment was fully replaced with THP-1 cell culture medium (RPMI) on day 12, and the effects on coculture monolayer integrity were evaluated by TEER measurement over the following 72 h. The medium switch did not cause any significant difference in TEER (Supplementary Figure S3).

Taken together, these results suggest that shortening the Caco-2/HT29-MTX coculture differentiation period from 21 to 14 days enables repeated pro-inflammatory treatment without affecting epithelium integrity or inflammatory response. Finally, in contrast to previous suggestions (Kampfer et al., 2017), the epithelial coculture can be fully switched to RPMI medium without any effect on coculture physiology in terms of tissue barrier integrity.

### THP-1 Differentiation Protocol

Previous studies have described several different THP-1 differentiation protocols, with great variation in PMA concentration, treatment duration, and inclusion or omission of resting period (Daigneault et al., 2010; Maess et al., 2014; Lund et al., 2016). To optimize laboratory timing and workload, we optimized a THP-1 differentiation protocol for over-the-weekend induction (∼65 h), thereby creating a wider time window for triculture assembly and compound assessment. Cells were seeded in a 24-well plate by adapting previous internally established 96-well plate format conditions (20,000 cells/well in 100 µl). In particular, 12 × 10^4^ cells were seeded in 600 µl of cell culture medium. Before stimulation of differentiated THP-1 cells with a pro-inflammatory inducer for an additional 24 h, a combination of different concentrations of PMA (10, 20, and 40 ng/ml) and post-differentiation resting times (24 and 48 h) were evaluated to assess the release of IL-8 and TNFα. LPS (10 ng/ml) was chosen as the pro-inflammatory inducer because LPS treatment had no effect on Caco-2/HT29-MTX cocultures (Supplementary Figures S2D–F), and, therefore, any effect on the epithelial coculture would be due to activation of the immune cells.

**FIGURE 2:**
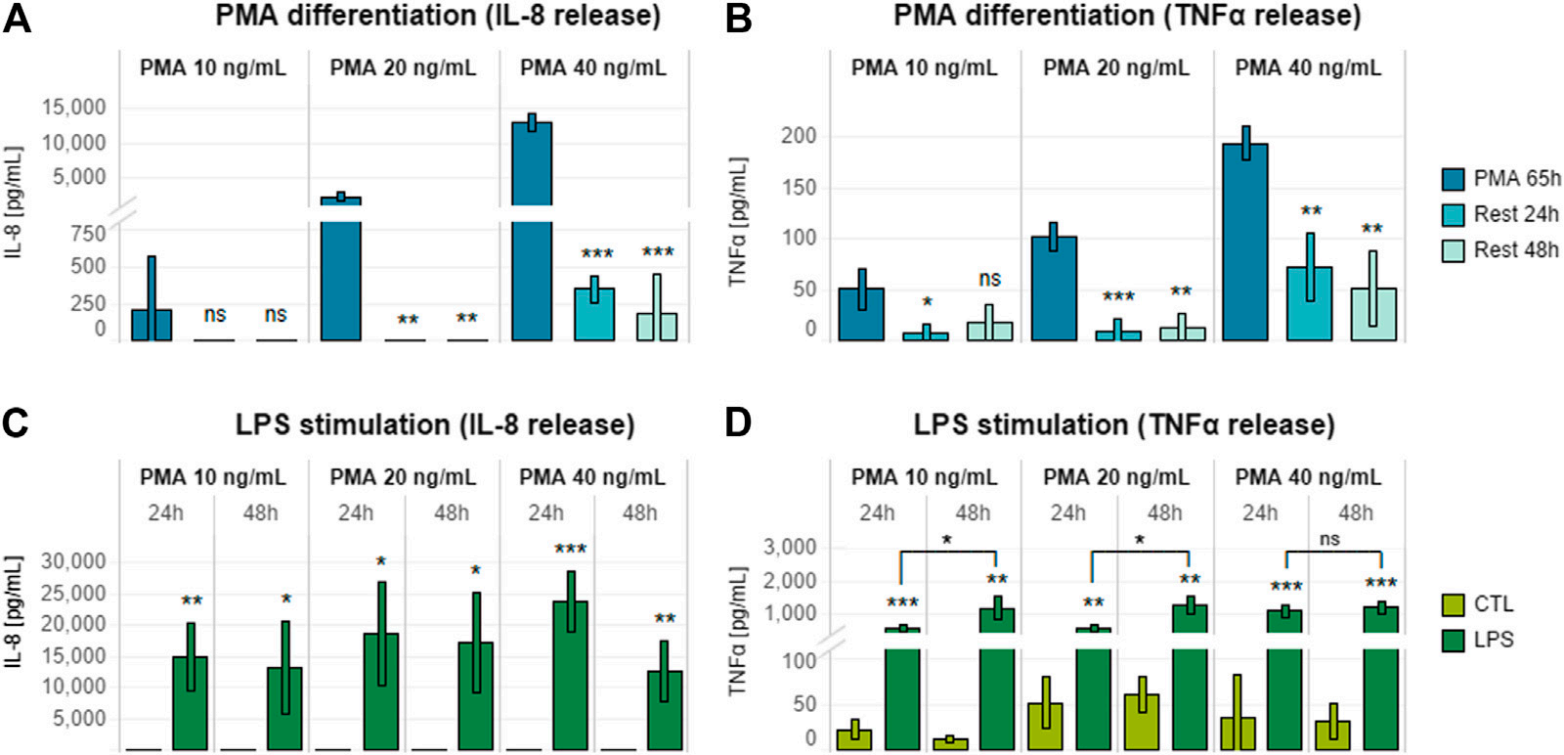
Effects of different PMA concentrations on THP-1 post-differentiation cytokine release. **(A)** IL-8 and **(B)** TNFα release by THP-1 cells differentiated for 65 h with increasing concentrations of PMA (10, 20, and 40 ng/ml) and rested for 24 or 48 h after PMA removal. **(C)** IL-8 and **(D)** TNFα release by THP-1 cells differentiated with increasing concentrations of PMA (10, 20, and 40 ng/ml) for 65 h, rested for 24 or 48 h after PMA removal, and treated with 10 ng/ml LPS for 24 h. Data are presented as the average of three technical replicates with corresponding standard deviation. Statistical differences were calculated between the 65-h induced sample and the rested samples **(A,B)** or between LPS-treated and untreated cultures **(C,D)** by means of two-tailed *t*-tests. *p ≤ 0.05; **p ≤ 0.01; ***p ≤ 0.001; ns, *p* > 0.05 **(A,B)**. Equivalence comparisons and linear regression analysis are provided in Supplementary Tables S3 and S4. R24h = cells rested for 24 h; R48h = cells rested for 48 h. Abbreviations: PMA, phorbol 12-myristate.

Increasing PMA concentrations caused a dose-dependent increase in both IL-8 and TNFα release by THP-1 cells (Figures 2A,B). PMA removal and 24 h of resting were required to obtain a significant decrease in the levels of both mediators; an additional 24-h rest did not result in any further decrease in IL-8 or TNFα levels. LPS treatment after 24 or 48 h of resting induced increased levels of IL-8 and TNFα release relative to untreated rested controls (Figures 2C,D). Interestingly, TNFα release was higher when a 48-h resting time was applied after stimulation with PMA (Figure 2D).

Taking into account the results of the LPS dose–response analysis, it was interesting to see that 48 h of resting was beneficial for achieving a greater pro-inflammatory response (Figure 2D). This aspect fitted well the concept of allowing 24 h of stabilization upon triculture assembly. In addition, we decided to exclude the differentiation with 40 ng/ml, as we observed no further increase in cytokines release relative to 10 or 20 ng/ml.

In order to choose the most appropriate condition for THP-1 maturation, differentiation with both 10 and 20 ng/ml PMA was further assessed in the context of the assembled triculture response.

### Coculture Assembly and Pro-Inflammatory Induction

THP-1 cells (12 × 10^4^) differentiated with 10 or 20 ng/ml PMA and rested for 24 h were assembled in a triculture with the Caco-2/HT29-MTX coculture on day 13, and the stability of the triculture was evaluated by TEER and permeability measurement. Neither endpoint decreased over the following 72 h (Supplementary Figures S4A,B). Following addition of a single concentration of LPS (10 ng/ml) to the basolateral side 24 h after triculture assembly, only small changes in TEER were observed in the subsequent 24 or 48 h (Supplementary Figure S5A), with no sign of increased permeability (Supplementary Figure S5B). These results confirmed that both concentrations of PMA were suitable for obtaining a stable triculture under pro-inflammatory induction.

**FIGURE 3:**
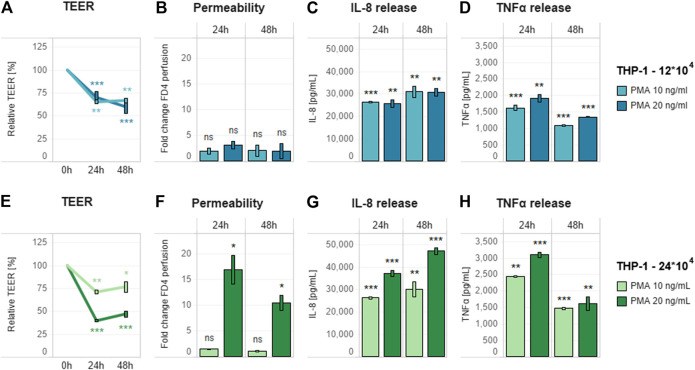
Effect of pro-inflammatory stimulation on optimized triculture. Pro-inflammatory effect of 10 ng/ml LPS added basolaterally to a triculture containing **(A–D)** 12 × 10^4^ and **(E–H)** 24 × 10^4^ THP-1 cells in 400 µl of cell culture medium in the basolateral compartment. **(A,E)** TEER **(B,F)** membrane permeability, and **(C,D,G,H)** cytokine release (IL-8 and TNFα, respectively) of the triculture. For each THP-1 seeding condition, different PMA concentrations (10 and 20 ng/ml) were tested for optimizing the triculture response. TEER values are represented in percentage; zero-hour treatment was used as the reference (100%). Permeability is expressed as fold change over the untreated control without LPS. Data are presented as the average of three technical replicates with the corresponding standard deviations. Statistical differences between the LPS- and PMA-treated cultures and the control culture without the compounds (with 12 × 10^4^ THP-1 cells) were determined by a two-tailed *t*-test. *p ≤ 0.05; **p ≤ 0.01; ***p ≤ 0.001; ns, *p* > 0.05. Abbreviations: PMA, phorbol 12-myristate; TNF, tumor necrosis factor; IL, interleukin; CTL, control.

However, they also suggested that the pro-inflammatory induction was not sufficient to affect the epithelial monolayer, thus reducing the applicability of these conditions for anti-inflammatory compound screening. This weak effect could be related to the levels of the released cytokines (Supplementary Figure S5B), especially TNFα, which is known to be the main factor responsible for the pro-inflammatory effects on the epithelial layer. To overcome these limitations and obtain a higher concentration of secreted TNFα, we first decided to decrease the cell culture medium volume in the basolateral compartment from 600 to 400 μl. Then, while assessing both concentrations of PMA (10 and 20 ng/ml), we also evaluated a higher number of THP-1 cells (24 × 10^4^). All selected conditions were assessed in the presence of the same previously tested pro-inflammatory stimulus (10 ng/ml LPS). Decreasing cell culture medium volume alone was not sufficient to induce an increase in epithelial layer permeability, because it only caused a decrease in TEER, as in the previous evaluation (Figures 3A,B). Differentiation of 2.4 × 10^5^ THP-1 cells in combination with 20 ng/ml PMA treatment appeared to be the most effective condition, as it caused the greatest decrease in TEER (Figure 3E) and increase in membrane permeability (Figure 3F). This combination also showed the highest level of cytokine release (Figures 3G,H).

**FIGURE 4:**
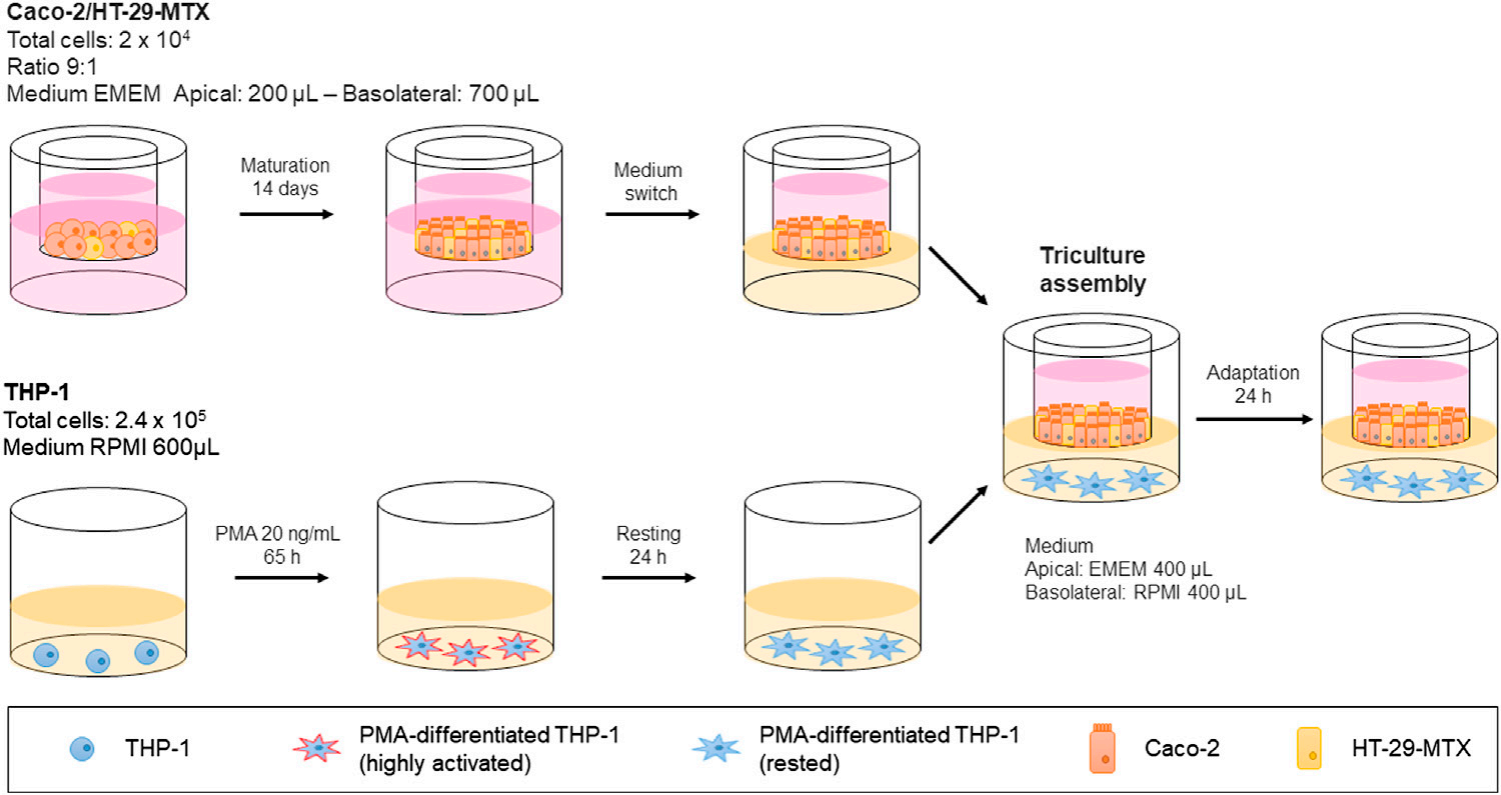
Schematic representation of the triculture assembly protocol.

With the conditions for triculture assembly (Figure 4) and proinflammatory induction defined, we continued by evaluating the suitability of the assay protocol for assessing anti-inflammatory properties by using known reference compounds. To this end, we chose budesonide and TPCA-1, because the former represents an effective drug that leads to remission in a subset of UC patients, while the latter is a known nuclear factor (NF)-κB pathway inhibitor.

### Reference Compound Assessment

The triculture was pretreated on the basolateral side for 6 h with different concentrations of selected reference compounds—TPCA-1 (0–10 µM) and budesonide (0–10 nM)—before addition of the pro-inflammatory stimulus (10 ng/ml LPS). After an additional 18 h (24-h time point) of incubation, TEER and monolayer permeability were assessed to evaluate epithelial monolayer integrity. In addition, IL-8 and TNFα release into the basolateral compartment was quantified, and cell viability was evaluated by WST-8 bioreduction separately in Caco-2-HT29-MTX cocultures and THP-1 cells (Supplementary Figure S6).

**FIGURE 5:**
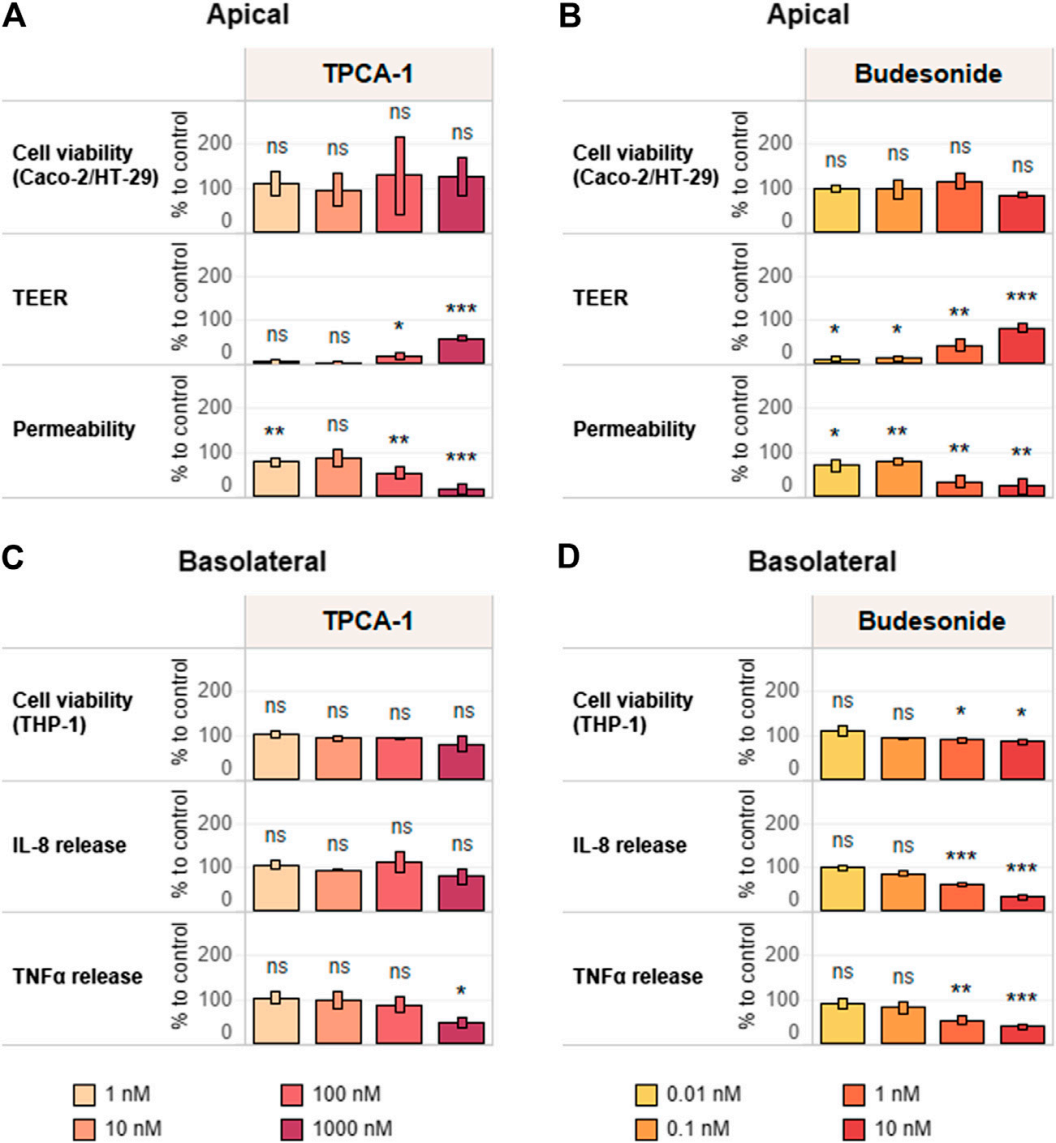
Reference item assessment and verification of the anti-inflammatory protocol. Tricultures were treated basolaterally with increasing concentrations of **(A,C)** TPCA-1 and **(B,D)** budesonide for 6 h and subsequently stimulated with 10 ng/ml LPS for 18 h. TEER, epithelial layer permeability, cell viability of differentiated Caco-2/HT29-MTX cells **(A,B)** and cytokine release and cell viability of differentiated THP-1 cells **(C,D)** were measured. TEER values are normalized to control (CTL; 100%) and LPS treatment (0%). Permeability is normalized by using LPS treatment as reference (100%). Basolateral IL-8 and TNFα release are expressed as percentage of control (cells treated with LPS only; CTL; 100%). Cell viability values of Caco-2-HT29-MTX cocultures and THP-1 cells are expressed as percentage of control (cells treated with LPS only, CTL; 100%). *n* = 3 independent experiments. Statistical differences between reference compound-treated vs. LPS-treated-only cultures were determined by a two-tailed *t*-test. *p ≤ 0.05; **p ≤ 0.01; ***p ≤ 0.001; ns, *p* > 0.05. Abbreviations: LPS, lipopolysaccharide; TEER, transepithelial electrical resistance; TNF, tumor necrosis factor; IL, interleukin.

Both TPCA-1 and budesonide, when compared against the LPS-treated-only control, induced dose–response changes in the investigated inflammation-related endpoints—that is, TEER and permeability—in the context of the epithelial monolayer. Pretreatment with these anti-inflammatory compounds prevented the LPS-induced effects on both TEER and membrane permeability (Figures 5A,B). The effects in the immune cell compartment appeared to be stronger upon pre-exposure to budesonide. In fact, the levels of both investigated cytokines were decreased in a dose-dependent manner (Figure 5D). On the other hand, treatment with TCPA-1 resulted in a decrease in TNFα levels only (Figure 5C). Finally, the absence of cytotoxicity in both cell compartments (apical: Figures 5A,B; basolateral: Figures 5C,D) proved the suitability of the established anti-inflammatory protocol for measuring the efficacy of anti-inflammatory compounds in a specific way.

### Alkaloid Assessment

**FIGURE 6:**
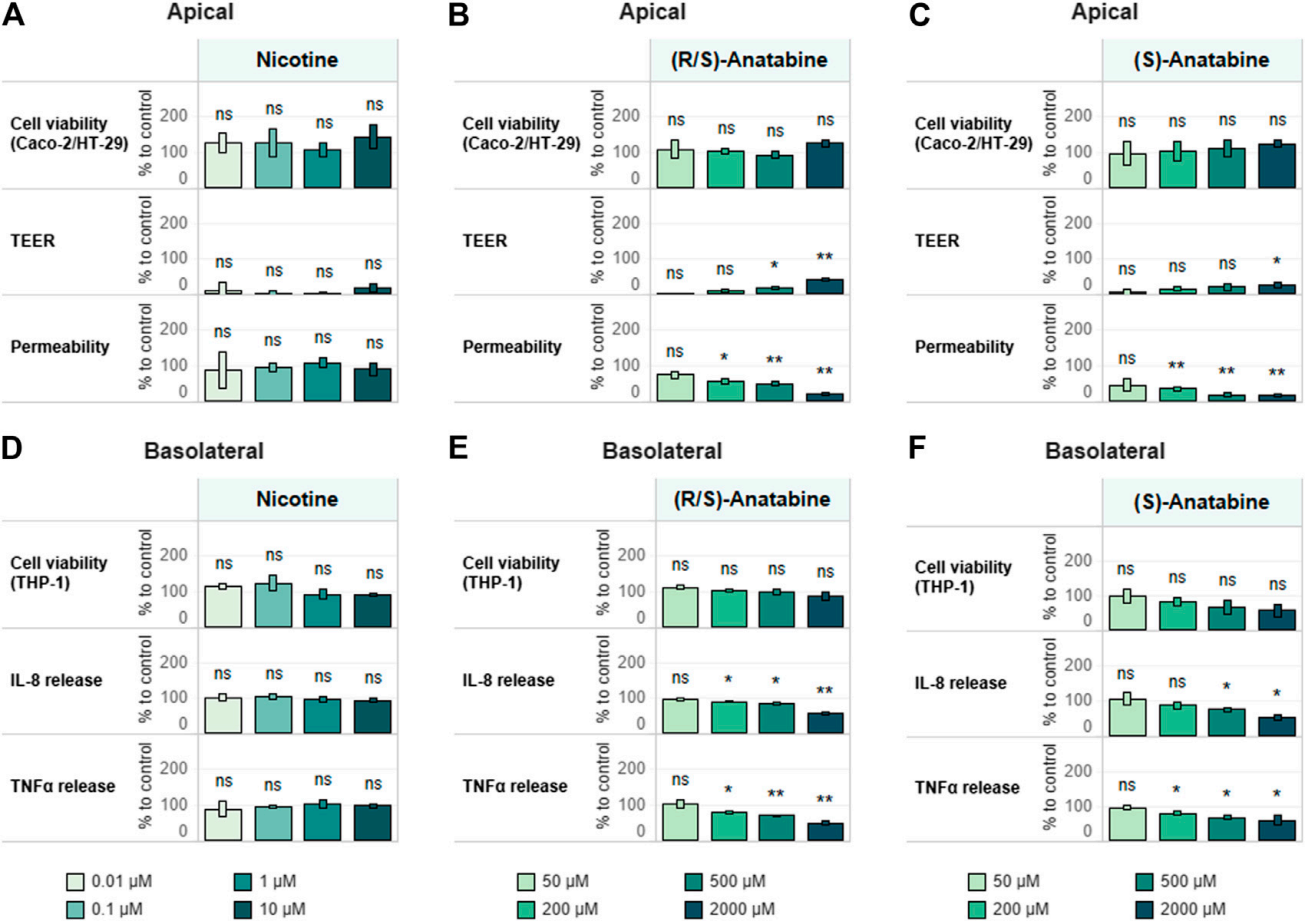
Test item assessment. Tricultures were treated basolaterally with increasing concentrations of **(A,D)** nicotine **(B,E)** (*R*/*S*)-anatabine, or **(C,F)** (*S*)-anatabine for 6 h and subsequently stimulated with 10 ng/ml LPS for 18 h. TEER, epithelial layer permeability, cell viability of differentiated Caco-2/HT29-MTX cells **(A–C)** and cytokine release and cell viability of differentiated THP-1 cells **(D–F)** were measured. TEER values are normalized to control (CTL; 100%) and LPS treatment (0%). Permeability is normalized by using LPS treatment as the reference (100%). Basolateral IL-8 and TNFα release are expressed as percentage of control (cells treated with LPS only; CTL; 100%). Cell viability values of Caco-2–HT29-MTX cocultures and THP-1 cells are expressed as percentage of control (cells treated with LPS only; CTL; 100%). *n* = 3 independent experiments. Statistical differences between the reference compound-treated vs. LPS-treated-only cultures were determined by a two-tailed *t*-test. *p ≤ 0.05; **p ≤ 0.01; ***p ≤ 0.001; ns, *p* > 0.05. Abbreviations: LPS, lipopolysaccharide; TEER, transepithelial electrical resistance; TNF, tumor necrosis factor; IL, interleukin; CTL, control.

Using the approach developed and verified with the reference compounds (Supplementary Figure S4), we assessed the anti-inflammatory properties of known tobacco alkaloids such as nicotine and anatabine. Of note, anatabine was tested as a citrate salts in both racemic (*R*/*S*) and *S* forms. Although the anti-inflammatory effects of nicotine have been previously described (Wang et al., 2003; de Jonge et al., 2005; Cloëz‐Tayarani and Changeux, 2007), we observed no such effect within the concentration range tested in this study (Figures 6A,D). Unlike nicotine, racemic anatabine showed concentration-dependent effects on both epithelial membrane integrity and TEER (Figure 6B). The observed effect is most likely due to an anti-inflammatory effect exerted on THP-1 cells, as evident from the decrease in cytokine release (Figure 6E). The S form exhibited comparable anti-inflammatory effects to those of the racemate. Nonetheless, S form-mediated effects appeared to be unspecific because the S form also induced greater cytotoxicity in the immune cells (Figures 6C,F). It is, in fact, reasonable to believe that the observed phenotype was caused by the decreased viability and functionality of the THP-1 cells. Comparison of the results of the racemic (*R*/*S*) and *S* forms of anatabine suggests that the *R* form is most likely less toxic and more specific with regard to anti-inflammatory effects.

## Discussion

In the present study, we established a biologically relevant intestinal *in vitro* system for assessing the potential anti-inflammatory effects of immunomodulating natural compounds. We leveraged previously established and published *in vitro* models (Ponce de León-Rodríguez et al., 2018) in which different intestinal epithelial cells were cultured in the presence or absence of immunocompetent cells. Although most features of the adaptive immune response are beyond the scope of current *in vitro* models, the features of innate immune response can be mimicked.

Although not fully representative of the true physiology of cells in their original tissues, immortalized cell lines still represent an adequate solution for profiling therapeutically active compounds. In fact, compared to primary cells, cell lines are characterized by an extended lifespan while still retaining some of their key functional features. The use of cell lines also offers the advantage of decreased experimental variability in screening approaches. For these reasons we selected the most commonly used epithelial cells, namely Caco-2 cells, complemented with mucus-secreting HT29-MTX cells (Huet et al., 1987; Lesuffleur et al., 1990) to better resemble the *in vivo* tissue counterpart. Caco-2 cells can respond to various cytokines or chemokines secreted by immune cells by modulating their membrane permeability, thus further amplifying or attenuating the inflammatory process (Jung et al., 1995; Panja et al., 1998; Bruewer et al., 2005; Weglarz et al., 2007; Hollebeeck et al., 2012; Rodriguez-Ramiro et al., 2013). Altogether, these features make Caco-2 cells one of the most accepted *in vitro* models of human enterocytes currently available.

Furthermore, the presence of mucus—secreted by HT29-MTX—in the cell system is essential for estimating intestinal permeability and adsorption (Mahler et al., 2009). In fact, it acts as a barrier against certain compounds, particularly those that are lipophilic (Behrens et al., 2001), preventing easy access of highly diffusible small molecules (Artursson et al., 2001). In the intestinal mucosa, the close localization of epithelial cells with immune cells with the ability to differentiate into macrophages or dendritic cells has prompted the hypothesis that their interactions are paramount for tissue homeostasis and, in some instances, might promote inflammation (Vitale et al., 2016). Consequently, we selected the monocyte-like cell line THP-1 for the immune component because of its capacity to be differentiated into macrophage- or dendritic cell-like cells.

Of note, the above-described characteristics do not allow us to exactly define which part of the intestine, small or large, this *in vitro* intestinal system may represent. In fact, although the differentiated Caco-2 cells form a polarized epithelial cell monolayer that resembles the enterocytes lining the small intestine, they have been shown to express both enterocyte and colonocyte genes (Grasset et al., 1984; Engle et al., 1998). Moreover, the presence of HT29-MTX cells further complicates the search for a definition, which may ultimately be considered forced. In fact, on the one hand, the HT29-MTX cells decrease the overall tightness of the monolayer, again pushing the model toward a small intestinal system (Hilgendorf et al., 2000); on the other hand, acting as goblet cells and being present at a fairly abundant level (9:1 ratio) (Hilgendorf et al., 2000; Chen et al., 2010), the HT29-MTX cells may better represent a condition closer to that in the large intestine. Thus, a more accurate and deeper characterization of the model at both molecular and physiological levels is necessary to clearly define it.

The triculture was generated with a dual-compartment geometry by using a Transwell® system, where the epithelial compartment is cultured on the apical side, and the immunocompetent cells are seeded on the basolateral side. The triculture assembly and stimulus protocol developed in this study allowed us to produce an *in vitro* model characterized by several advantages: i) less laborious cell culture handling, which did not require adaptation of cell culture medium, use of extracellular matrix, or pro-inflammatory cell priming; ii) direct immune-to-epithelial cell pro-inflammatory activation, as the selected pro-inflammatory stimulus selectively activated the immune cells, which further extended the effect to the epithelial component; iii) a set of functional readouts that well recapitulate the *in vivo* phenotype; and iv) feasibility for multiple cycles of pro-inflammatory stimulus for assessing repeated treatment.

We also proved the suitability of the model for use as a screening tool for anti-inflammatory compounds by assessing two known anti-inflammatory drugs, both of which successfully prevented LPS-induced pro-inflammatory effects in a dose-dependent manner. The model also allowed further characterization of known tobacco alkaloids, such as nicotine and anatabine, which have previously been shown to have anti-inflammatory abilities in animal models of intestinal diseases (Paris et al., 2013b; Zabrodskii et al., 2015; Maruta et al., 2018). Several studies have reported nicotine-dependent anti-inflammatory effects in animal models of IBD (Maruta et al., 2018). In Caco-2 cells, nicotine has been shown to up-regulate the expression of the tight junction proteins occludin and claudin-1, and improve barrier function, followed by anatabine and the primary metabolite of nicotine cotinine, although to a lower extend (McGilligan et al., 2007). Conversely, nicotine showed no such effects in our *in vitro* intestinal model, highlighting the importance of using *in vitro* models that replicate key features of the intestinal mucosa for compound testing. On the other hand, the administration route of nicotine *in vivo* models (i.e., oral administration, subcutaneous injection) plays a crucial role in the potential anti-inflammatory effects of nicotine in intestinal inflammation (AlSharari et al., 2013) therefore we might have not been able to capture the full potential of nicotine with our model as nicotine dissemination through the blood stream, intestinal absorption, or gastrointestinal metabolism might play a crucial role in nicotine bioavailability and anti-inflammatory effects in the gut.

Of note, other minor tobacco alkaloids, nAChR agonists as well, were also suggested as having potential anti-inflammatory effects (Olsson et al., 1993; Bai et al., 2007; Alijevic et al., 2020; Ruiz Castro et al., 2020). Among these, the alkaloids anatabine and cotinine have displayed protective effects in animal models of inflammatory conditions, including Alzheimer’s disease (Paris et al., 2011), Parkinson’s disease (Barreto et al., 2014), sepsis (Zabrodskii et al., 2015), and IBD (Bai et al., 2007; Ruiz Castro et al., 2020). Our results on anatabine are thus substantiated by the findings of previous studies that have demonstrated the anti-inflammatory effects of anatabine in *in vivo* disease models, indicating that this pyridine alkaloid might be a potential candidate for development of anti-inflammatory therapies (Paris et al., 2011; Paris et al., 2013b).

In summary, our results show that our *in vitro* triculture intestinal model exhibits mucosal immune responses and stable barrier function analogous to those *in vivo* and is suitable for screening compounds with anti-inflammatory properties. By capturing the key features of intestinal inflammation, this *in vitro* tool provides a means to investigate immunomodulating therapeutic intervention of the inflamed intestine.

## Data Availability

The original data presented in the study are included in the article/Supplementary Material. Further inquiries can be directed to the corresponding author.
