# Supplementary material for: Development of an Advanced Multicellular Intestinal Model for Assessing Immunomodulatory Properties of Anti-Inflammatory Compounds
Source: Front Pharmacol. 2021 Apr 16;12:639716. doi: 10.3389/fphar.2021.639716 (PMC8085553; doi:10.3389/fphar.2021.639716)
Supplement: Supplementary file 1 [file datasheet1.pdf]

Supplementary Material

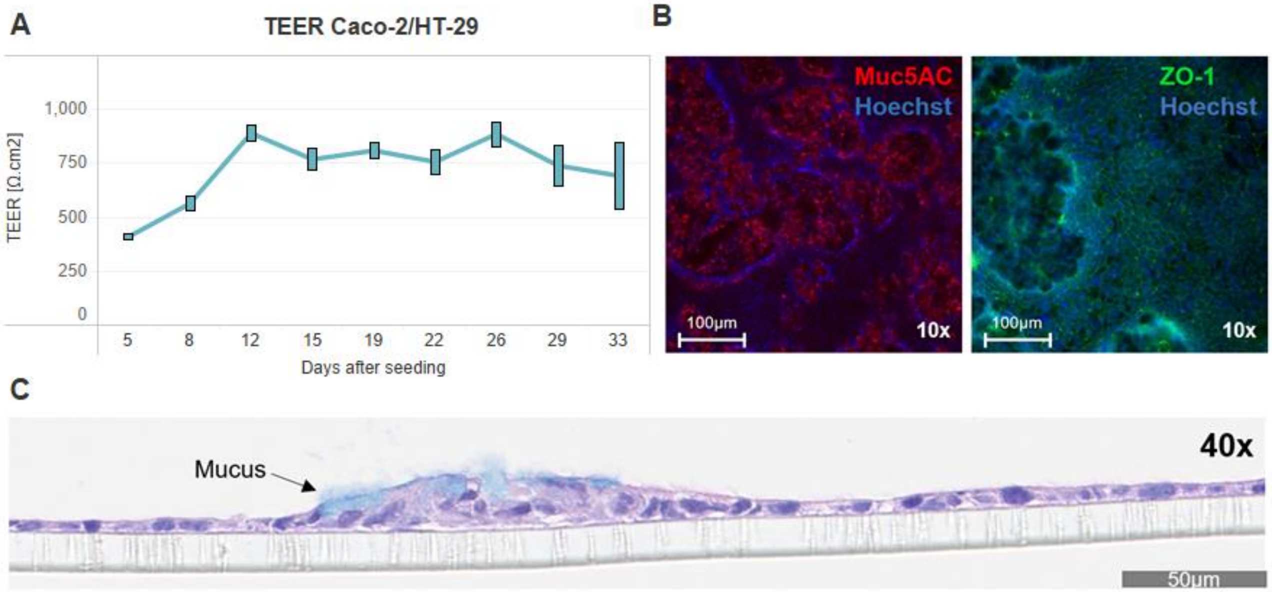

Supplementary Figure 1. Caco-2/HT29-MTX coculture differentiation. A) Caco-2/HT29-MTX coculture TEER profile measured from the day 100% cell confluence was reached up to day 32. Average value and corresponding standard deviation obtained from 24 individual trans-wells. (B) Immunofluorescence staining for Muc5AC and ZO-1, both combined with Hoechst, on day 12. C) Representative image of hematoxylin, eosin, and Alcian blue-stained culture sections obtained on day 12. Abbreviations: Muc, mucin; TEER, trans epithelial electrical resistance; ZO, zona occludens.

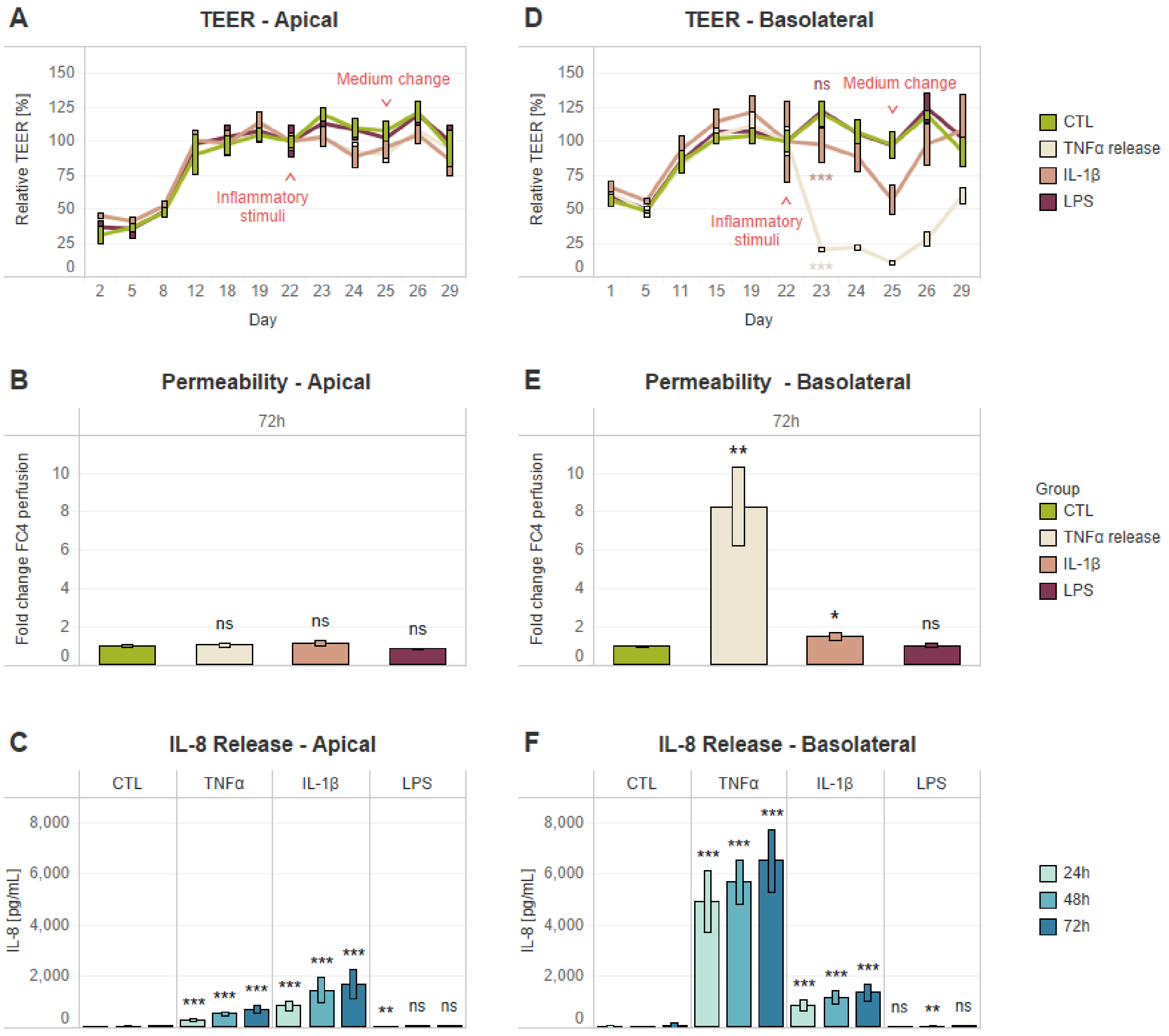

Supplementary Figure 2. Optimization of induction of inflammation in Caco-2/HT29 monoculture. TEER (**A** and **D**), permeability (**B** and **E**), and IL-8 release (**C** and **F**) in differentiated Caco-2/HT29 cocultures stimulated apically (**A–C**) or basolaterally (**D–F**) with inflammatory inducers (100 ng/mL TNFα, 100 ng/mL IL-1β, or 50 µg/mL LPS) on day 22. The inflammatory stimuli were left up to day 25(72 h) and then washed out. TEER and IL-8 release was assessed at 24, 48 and 72 h after pro-inflammatory stimuli were added while permeability was only assessed after 72 h. TEER values are expressed in percentage; measurements obtained before addition of inflammatory stimuli (day 25) were used as reference (100%). Permeability is represented as fold change over the untreated control. Data are presented as average of 4 individual trans-wells with their corresponding standard deviation. Statistical differences between the control and each of the three treatments were determined by a two-tailed t-test, $*p \leq 0.05$; $**p \leq 0.01$; $***p \leq 0.001$; ns, $p > 0.05$. Abbreviations: LPS, lipopolysaccharide; TEER, trans epithelial electrical resistance; TNF, tumor necrosis factor; IL, interleukin; CTL, control.

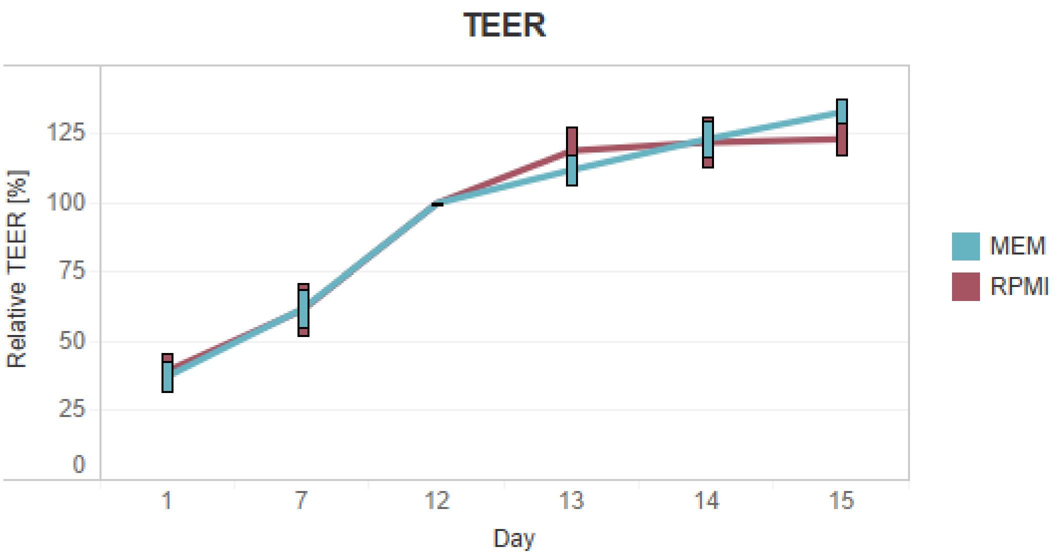

Supplementary Figure 3. Caco-2/HT29-MTX coculture medium compatibility. TEER measurement in Caco-2/HT29-MTX coculture switched to THP-1 medium (RPMI) for 72 h on day 12. CTL was maintained in regular coculture medium (MEM). TEER is expressed as relative percentage, with the value on day 12 used as reference (100%). Data are presented as average of 12 individual trans-wells with their corresponding standard deviation. Abbreviations: TEER, trans epithelial electrical resistance.

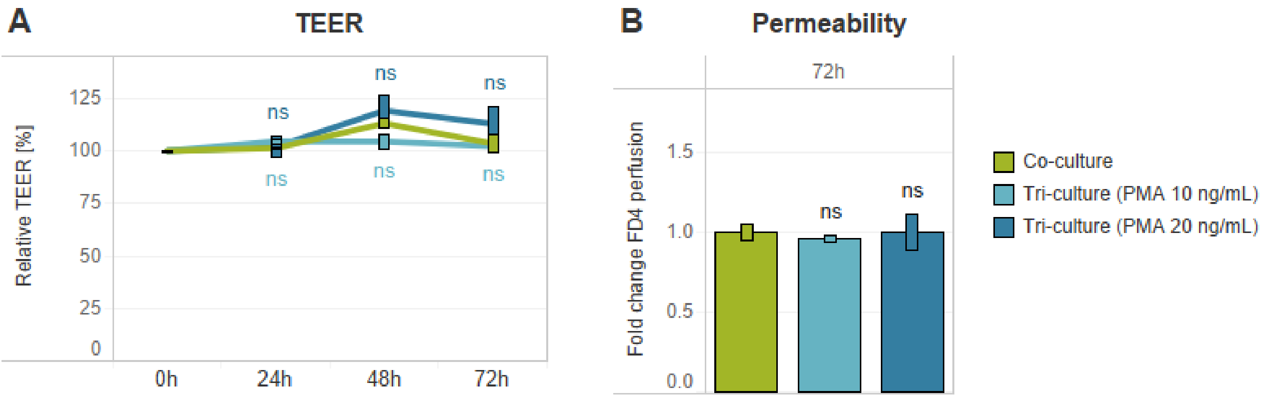

Supplementary Figure 4. Caco-2/HT29-MTX/THP-1 triculture assembly. (**A**) TEER measurement in Caco-2/HT29-MTX coculture was measured every day up to 72 h after triculture assembly. TEER is expressed as relative percentage, with the value on the time of assembly (0h) used as reference (100%). (**B**) Permeability assessed 72 h after triculture assembly. Permeability is represented as fold change over Caco-2/HT29-MTX coculture. Data are presented as average of 2 individual trans-wells with their corresponding standard deviation. Statistical differences between the PMA-treated tri-culture and the co-culture were determined by a two-tailed t-test, $*p \leq 0.05$; $**p \leq 0.01$; $***p \leq 0.001$; ns, $p > 0.05$. Abbreviations: PMA, phorbol-12-myristate-13-acetate; TEER, trans epithelial electrical resistance.

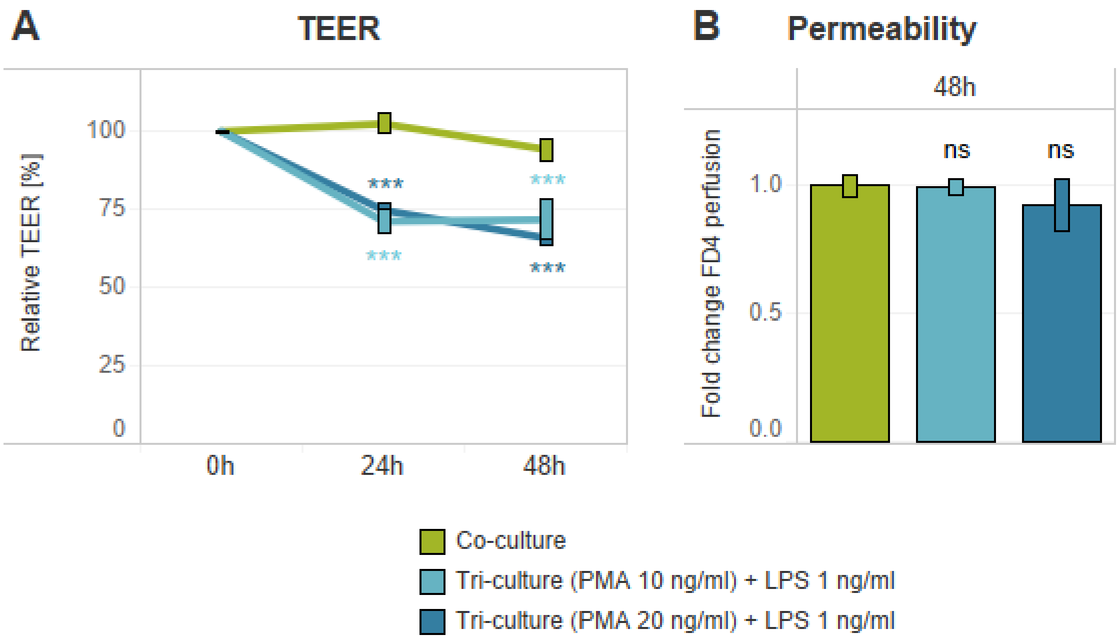

Supplementary Figure 5. Effect of pro-inflammatory stimulation on triculture. Pro-inflammatory effect of 10 ng/mL LPS added basolaterally on 12 x 10^4 THP-1 containing triculture with a volume of 600 µL of cell culture media in the basolateral compartment. (**A**) TEER, (**B**) membrane permeability, and (**C**) IL-8 release of the triculture. Different PMA concentrations (10 and 20 ng/mL) were tested for optimizing the triculture response. TEER values are represented in percentage; zero-hour treatment was used as reference (100%). Permeability is expressed as fold change over the untreated control. Data are presented as the average of two technical replicates with corresponding standard deviation. Statistical differences between the LPS- and PMA-treated culture and the co-culture without them were determined by a two-tailed t-test, $*p \leq 0.05$; $**p \leq 0.01$; $***p \leq 0.001$; ns, $p > 0.05$. Abbreviations: PMA, phorbol 12-myristate; TNF, tumor necrosis factor.

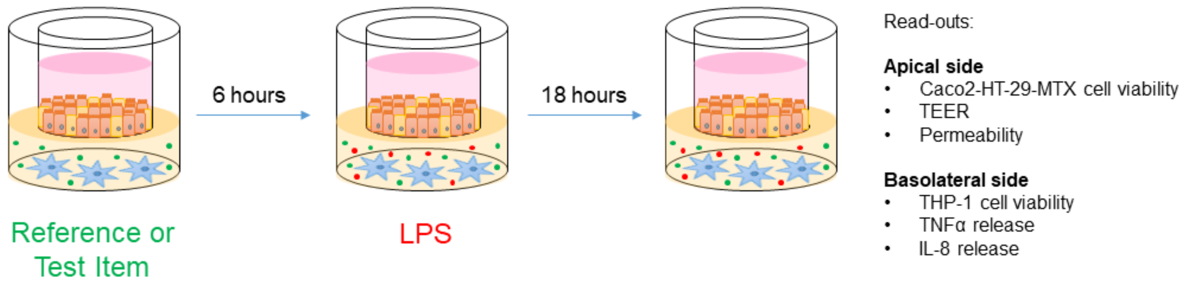

Supplementary Figure 6. Schematic representation of the anti-inflammatory screening protocol. Reference or test items are preincubated in the basolateral compartment for 6 h before the pro-inflammatory stimulus (LPS) is added to the same compartment. After an additional 18 h of coincubation, the indicated readouts are obtained. Abbreviations: LPS, lipopolysaccharide.

Supplementary Table 1. Equivalence comparison between 21 and 14 day protocol (figure 1).

Symbols and abbreviations: ***: $p \leq 0.001$; ns: $p > 0.05$.

		21 vs 14			
**Panel**	**Endpoint**	**5 mg/ml**	**25 mg/ml**	**100 mg/ml**	*Epsilon*
A & B	TEER	***	***	***	*20%*
C	Permeability	***	***	***	*20%*
D	IL-8 apical	n.s.	n.s.	n.s.	*20%*
E	IL-8 basal	n.s.	n.s.	n.s.	*20%*

Supplementary Table 2. Linear regression TEER recovery (figure 1).

Symbols and abbreviations: ***: $p \leq 0.001$

Panel	Endpoint	5 mg/ml	25 mg/ml	100 mg/ml
A	TEER	***	***	***
B	TEER	***	***	***

Supplementary Table 3. Equivalence comparison between 24 h and 48 h resting time protocol (figure 2).

Abbreviations: n.s., not significant, n.c., not computable

		24 h vs 48 h			
**Panel**	**Endpoint**	**PMA 10 ng/ml**	**PMA 20 ng/ml**	**PMA 40 ng/ml**	*Epsilon*
A	IL-8	n.c.	n.c.	n.s.	*20%*
B	TNFα	n.s.	n.s.	n.s.	*20%*
C	IL-8	n.s.	n.s.	n.s.	*20%*
D	TNFα	n.s.	n.s.	n.s.	*20%*

Supplementary Table 4. Linear regression PMA concentration (figure 2).

Symbols and abbreviations: ***: $p \leq 0.001$

Panel	Endpoint	PMA
A	IL-8	***
B	IL-8	***

Supplementary Table 5. Product information of reference compounds, test compounds, and pro-inflammatory inducers

	**Short name**	**Manufacturer (Cat #)**	**CAS number**
Reference compounds	Budesonide	Sigma B7777	51333-22-3
	TPCA-1	Tocris	507475-17-4
	Nicotine	Sigma N3876-100mL	54-11-5
Test compounds	(*R*/*S*)-Anatabine	Concept Life Sciences	2022507
	(*S*)-Anatabine	Peakdale Molecular Ltd	2022509
Inducers	TNFα	R&D Systems (210-TA)	NA
	IL-1β	R&D Systems (201-LB)	NA
	LPS	Sigma (L2630)	NA
